# Factors associated with early progression of non-small-cell lung cancer treated by epidermal growth factor receptor tyrosine-kinase inhibitors

**DOI:** 10.1002/cam4.180

**Published:** 2014-01-10

**Authors:** Nathalie Rozensztajn, Anne-Marie Ruppert, Armelle Lavole, Etienne Giroux Leprieur, Michael Duruisseaux, Thibault Vieira, Nathalie Rabbe, Roger Lacave, Martine Antoine, Jacques Cadranel, Marie Wislez

**Affiliations:** 1 Service de Pneumologie, Hôpital TenonAP-HP, 4 rue de la Chine, Paris, 75970, France; 2 Equipe de Recherche2/GRC-UPMC04 Theranoscan, Université Pierre et Marie Curie Paris 6, Hôpital Tenon4 rue de la Chine, Paris, France; 3 Plateforme de Biologie moléculaire, Service de Cytologie et Biologie Tumorale, Hôpital TenonAP-HP, 4 rue de la Chine, Paris, 75970, France; 4 Service d'Anatomie Pathologique, Hôpital TenonAP-HP, 4 rue de la Chine, Paris, 75970, France

**Keywords:** Epidermal growth factor receptor tyrosine-kinase inhibitors, erlotinib, gefitinib, non-small-cell lung cancer, progressive disease

## Abstract

Epidermal growth factor receptor tyrosine-kinase inhibitors (EGFR-TKI) are a therapeutic option as second-line therapy in non-small-cell lung carcinoma (NSCLC), regardless of the *EGFR* gene status. Identifying patients with early progression during EGFR-TKI treatment will help clinicians to choose the best regimen, TKI or chemotherapy. From a prospective database, all patients treated with gefitinib or erlotinib between 2001 and 2010 were retrospectively reviewed. Patients were classified into two groups according to their tumor response by RECIST after 45 days of treatment, progressive disease (PD) or controlled disease (CD). Two hundred and sixty-eight patients were treated with EGFR-TKI, among whom 239 were classified as PD (*n* = 75) and CD (*n* = 164). Median overall survival was 77 days (95% CI 61–109) for PD and 385 days (95% CI 267–481) for CD. Patients with PD were of younger age (*P *=* *0.004) and more frequently current smokers (*P *=* *0.001) had more frequently a performance status ≥2 (*P *=* *0.012), a weight loss ≥10% (*P *=* *0.025), a shorter time since diagnosis (*P *<* *0.0001), a pathological classification as non-otherwise-specified NSCLC (*P *=* *0.01), and the presence of abdominal metastases (*P *=* *0.008). In multivariate analysis, abdominal metastases were the only factor associated with early progression (odds ratio (OR) 2.17, 95% CI [1.12–4.19]; *P *=* *0.021). Wild-type *EGFR* versus mutated *EGFR* was associated with early progression. The presence of abdominal metastasis was independently associated with early progression in metastatic NSCLC receiving EGFR-TKI.

## Introduction

Gefitinib and erlotinib are oral epidermal growth factor receptor (EGFR) tyrosine-kinase inhibitors (TKI). They are therapeutic options for metastatic non-small-cell lung cancers (NSCLC). In first-line settings, their use is restricted to patients whose tumor harbors an activating mutation of *EGFR*. For subsequent treatment lines, there is no such restriction, and the choice between EGFR-TKI and cytotoxic therapy is still debated.

Studies that address this matter are scarce. In unselected population with unknown EGFR mutation status, three studies have compared gefitinib to docetaxel (INTEREST, 1466 patients 1; V-15-32, 490 patients 2; Lee et al. 3, 164 patients), one study compared erlotinib to pemetrexed (Vamvakas et al. 4, 327 patients), one more recent study compared erlotinib to docetaxel or pemetrexed (TITAN, 424 patients 5), though in the setting of patients refractory to a platin-based chemotherapy doublet. These studies showed an equivalent efficacy of EGFR-TKI to chemotherapy on overall survival (OS) or time to progression, except for Lee et al.'s study, in which progression-free survival (PFS) was better with gefitinib. It can be hypothesized, particularly in Asian studies, that the benefit observed in the EGFR-TKI treatment arms was mainly driven by *EGFR*-mutated tumors. However, in these studies, in a small subset of patients for whom EGFR status was known and wild-type, efficacy was similar between the two treatment arms (26 patients in V15-32, 149 patients in TITAN, 253 patients in INTEREST). In contrast, in a recent phase III study in 222 patients selected with wild-type *EGFR* status (TAILOR, Garassino et al. 6), docetaxel did better than erlotinib as second-line therapy in terms of OS (8.2 vs. 5.4 months, HR 0.73 [0.53–1.00], *P* = 0.05) and PFS, (2.9 vs. 2.4 months HR 0.71 [0.53–0.95], *P* = 0.02). In another phase III study, the DELTA trial (Okano 7), the subset of patients with EGFR wild-type tumors had a shorter PFS (1.3 vs. 2.9 months, HR 1.44 [1.08–1.92], *P* = 0.013) with erlotinib than with docetaxel, but the OS did not differ significantly according to the treatment arm.

It seems that there are several subsets of patients with wild-type *EGFR* NSCLC, and that only some benefit from EGFR-TKI treatments. It is important to identify these subsets in order to choose the best therapeutic strategy. Although the clinical, pathological, and molecular markers that can predict a response to EGFR-TKI therapy are now well-known 1,8–20, no studies have searched potential markers associated with early progression versus disease control under these treatments 21. Because the proportion of patients with *EGFR-*mutated tumors, who represent the majority of “EGFR-TKI responding patients”, did not exceed 16.6% 22 in a Western European population, further information on markers of early progression could help clinicians to choose the best regimen, TKI or chemotherapy. The PROSE trial (Sorlini 23) demonstrates the feasibility of using the proteomic classifier Veristrat as a predictive tool, which could be useful in this indication. In this study, characteristics of patients who experience early progression were compared to patients whose disease was controlled. The aim was to identify characteristics associated with early progression with EGFR-TKI therapy.

## Patients and Methods

### Patients

All patients diagnosed with lung cancer are registered in a prospective database. All consecutive metastatic NSCLC registered in the database between 10-2001 and 05-2010 were reviewed. Patients that received erlotinib or gefitinib for at least 7 days and were evaluated for tumor response were included in this study. We chose 7 days because this is the time needed for EGFR-TKI to achieve stable plasma concentration.

Exclusion criteria were: presence of extrapulmonary malignancies (except localized prostate cancer treated by hormone-therapy, breast cancer under adjuvant hormone-therapy, stage A chronic lymphatic leukemia); concomitant carcinological treatment (chemotherapy, radiotherapy on the target lesion); and treatment interruption for >15 days before the first tumoral evaluation.

### Definition of early progression

Progression was defined according to RECIST criteria on tomodensitometry or magnetic resonance imaging (MRI) or on clinical progression when unequivocal (subcutaneous nodules, superficial lymph nodes, spinal cord compression). When this classification remained unsure, the patient was excluded from the comparative analyses. Cancer-related death was considered as progression. Early progression was defined as progression before the 45th day of treatment.

### Data collection

All patients presented histological proven NSCLC according to the World Health Organization guidelines and tumor stage was defined by the 7th TNM classification.

For each tumor, the following characteristics were analyzed: histological type and subtypes according to the 2004 WHO classification, mucin secretion using periodic acid-Schiff diastase staining, *EGFR* and *KRAS* mutations using PCR sequencing and *EML4-ALK* translocations by immunohistochemistry.

For each patient, the following characteristics were collected: age, gender, ethnic origin, smoking status (non smoker, former smoker, and current smoker), performance status (PS) according to the ECOG classification, weight loss since the time of diagnosis, presence and location of metastatic sites at the time of treatment initiation. The metastatic sites were separated into five categories: central nervous system metastasis (brain and leptomeninges), thoracic metastasis (lung, pleura, and pericardium), abdominal metastasis (liver, adrenal glands, spleen, pancreas, kidney, ovary, subdiaphragmatic lymph node, peritoneal carcinosis…), skin metastasis, and bone metastasis. The lack of data did not enable us to make a relevant analysis based on the characteristics of the bone metastasis: lytic or osteoblastic.

The other data assessed were: prior chemotherapy regimen, time from diagnosis to EGFR-TKI treatment, treatment toxicities, and vital status at date of end point (death, alive, or lost for follow-up).

### Statistical analyses

Statistical analyses for comparisons between groups were performed using the chi-squared test or Fisher's exact test for qualitative variables, and Student's *t*-test or the Mann–Whitney test for continuous variables. Variables that differed significantly (*P *<* *0.05) between the groups were included in a logistic regression model for univariate and multivariate analyses. Overall survival and PFS times from the beginning of EGFR-TKI treatment were analyzed using the Kaplan–Meier method. The log-rank test was used to test for differences in OS between the groups. Date of End point is May 31st, 2010.

## Results

**Figure 1:**
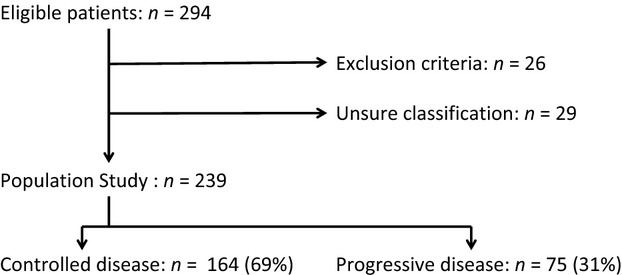
Flow chart of the 294 eligible patients.

Between 10-2001 and 05-2010, 294 eligible patients were identified. Fifty-five patients were excluded: 26 because they met the exclusion criteria and 29 because their classification into progressive disease (PD) or controlled disease (CD) was unsure (Fig. 1). The comparative analyses between the two groups were based on the 239 remaining patients. Seventy five patients (31.4%) were classified into the PD group, whereas 164 patients (68.6%) were classified into the CD group.

### Clinical and pathological characteristics

Mean age was 62.4 years. Most patients were male (60.7%), smokers (81.2%), but had stopped tobacco before the beginning of treatment (87.1%), and had a good PS (65.6%). Most patients (*n *=* *207, 86.6%) received chemotherapy prior to EGFR-TKI therapy and 192 (80.3%) received a platinum-based chemotherapy doublet (Table 1).

**Table 1:** Clinical and pathological characteristics at the time of epidermal growth factor receptor tyrosine-kinase inhibitors (EGFR-TKI) initiation

	Overall population (*n *=* *239)	Progressive disease group (*n *=* *75)	Controlled disease group (*n *=* *164)	*P*
Mean age (SD)^1^	62.4 (11.1)	59.4 (11.6)	63.8 (10.6)	0.004
Gender (%)^2^				
Women	94 (39.3)	28 (37.3)	66 (40.2)	0.669
Men	145 (60.7)	47 (62.6)	98 (59.8)	
Ethnic group (%)^3^				
Asian	10 (4.2)	1 (1.3)	9 (5.5)	0.137
Other	229 (95.8)	74 (98.7)	155 (94.5)	
Smoking status (lifelong; %)^2^				
Never-smoker	45 (18.2)	12 (16.0)	33 (20.1)	0.581
Current or ex-smoker	194 (81.2)	63 (84.0)	131 (79.9)	
Tobacco use (during EGFR-TKI treatment; %)^2^				
Ongoing	25 (10.5)	15 (20.0)	10 (6.1)	0.001
Stopped or never smoked	205 (85.8)	56 (74.7)	149 (90.9)	
Weight loss since diagnosis (%)^2^				
<10%	125 (52.3)	35 (46.7)	90 (54.9)	0.025
≥10%	33 (13.8)	16 (21.3)	17 (10.4)	
Performance status (%)^2^				
0 or 1	147 (61.5)	37 (49.3)	110 (67.0)	0.012
≥2	77 (32.2)	32 (42.7)	45 (27.4)	
Prior regimen (%)^2^				
None	32 (13.4)	10 (13.3)	22 (13.4)	0.993
One	84 (35.1)	26 (34.7)	58 (35.4)	
Two or more	123 (51.4)	39 (52.0)	84 (51.2)	
Presence of metastases (%)^2^				
Yes	218 (91.2)	69 (92.0)	149 (90.9)	0.771
No	21 (8.8)	6 (8.0)	15 (9.1)	
Metastatic site (%)^2^				
Thorax	140 (64.2)^4^	45 (65.2)^4^	95 (63.8)^4^	0.883
Abdomen	71 (32.6)^4^	31 (44.9)^4^	40 (26.8)^4^	0.008
Brain and meninges	55 (25.2)^4^	19 (27.5)^4^	36 (24.2)^4^	0.594
Bone	76 (34.9)^4^	24 (34.8)^4^	52 (34.9)^4^	0.897
Skin^3^	9 (4.1)^4^	6 (4.3)^4^	3 (4.0)^4^	1.0
Pathological type (%)^2^				
Adenocarcinoma	134 (56.1)	36 (48.0)	98 (59.8)	0.010
Squamous cell	39 (16.3)	9 (12.0)	30 (18.3)	
Nos-NSCLC	49 (20.5)	25 (33.3)	24 (14.6)	
Other	17 (7.1)	5 (6.7)	12 (7.3)	

Missing data have been suppressed. nos, non-otherwise-specified.

1 Student's *t*-test.

2 Chi-squared test.

3 Fisher's exact test.

4 Among stage IV patients (*n *=* *218).

The predominant pathologic type was adenocarcinoma (56.1%), followed by non-otherwise-specified (nos)-NSCLCs (20.5%), squamous cell carcinomas (16.3%) and other histological types (7.1%; Table 2). Histological subtypes were known for only 51.5% of adenocarcinoma, and the presence or absence of mucosecretion for 44.8% of adenocarcinoma (Table S1, supplementary data).

**Table 2:** Molecular characteristics of the 239 classified tumors

	Overall population (*n *=* *239)	Progressive disease group (*n *=* *75)	Controlled disease group (*n *=* *164)	*P*
*EGFR* gene (%)^1^				
Wild-type	82 (34.3)	29 (36.7)	53 (32.3)	0.002
Mutated	19 (7.9)	0 (0)	19 (11.6)	
Unknown^3^	138 (57.8)	46 (61.3)	92 (56.1)	
*KRAS* gene (%)^1^				
Wild-type	102 (42.7)	33 (44.0)	69 (42.1)	0.531
Mutated	9 (3.8)	2 (2.7)	7 (4.3)	
Unknown^3^	128 (53.6)	40 (53.3)	88 (53.6)	
*EML4-ALK* translocation (%)^2^				
Presence	4 (1.7)	0 (0)	4 (2.4)	0.293
Absence	38 (15.9)	13 (17.3)	25 (15.2)	
Unknown^3^	197 (82.4)	62 (82.7)	135 (82.3)	

1 Chi-squared test.

2 Fisher's exact test.

3 Missing data have been suppressed for the statistical analyses.

*EGFR*- and *KRAS*-gene status was known for 102 (42.7%) and 111 (46.4%) patients, respectively (Table 2). *EML4-ALK* translocation detection was done for 42 (17.5%) patients. *EGFR* gene mutations were detected in 19 tumors (7.9%). *KRAS*-gene mutation and *EML4-ALK* translocation were infrequent (3.8% and 1.7%).

Progression-free survival times were known for 208 patients; the data for 27 patients were censored. For the four remaining patients, there were missing data, but the PFS time was longer than 45 days. The median PFS was 80 days (95% CI 68–90). Vital status was known for 174 patients. Median OS was 242 days (95% CI 180–293).

### Factors associated with early progression during EGFR-TKI therapy

Several clinical characteristics were more frequent in the PD group: younger age (*P *= 0.004), current smoking (*P *= 0.001), PS ≥ 2 (*P *= 0.008), and weight loss ≥10% (*P *= 0.025).

There was no significant difference between the PD and CD groups concerning the presence of one or several metastases at the time of initiating EGFR-TKI therapy (Table 1). The distribution of metastatic locations was different between the PD and CD groups. Abdominal metastases were more frequent in the PD group (44.9% vs. 26.8%, *P *= 0.008). The distribution of abdominal metastasis is detailed in Table S2, supplementary data.

The predominant histological pattern in both PD and CD groups was adenocarcinoma (56.1%) followed by nos-NSCLCs (20.5%). Nos-NSCLC was more frequent in the PD group than in the CD group (33.3% vs. 14.6%, *P *= 0.01).

No *EGFR* gene mutation was detected in the PD group and *EGFR* gene mutations were detected in 19 tumors from patients in the CD group (11.6%; *P *= 0.002). *KRAS*-gene mutation and *EML4-ALK* translocation were infrequent and their distribution was not significantly different between the two groups (Table 2).

No significant difference on chemotherapy—prior to EGFR-TKI treatment—was noted between the groups, PD versus CD. There was no significant difference regarding the number of previous treatment lines between the groups (*P* = 0.993; Table 1). The median number of lines of chemotherapy received before the EGFR-TKI was 2 1,2 in both groups. The number of patients considered refractory to chemotherapy did not vary significantly between the groups: they were 12 in the PD group (16.0%) and 18 in the CD group (12.7%, *P *= 0.261). The same observation was made when only platinum-based therapy was considered, with 15 (20.0%) refractory patients in the PD group and 14 (11.7%) refractory patients in the CD group (*P *= 0.067). In contrast, the time from diagnosis to EGFR-TKI treatment was shorter in the PD group (median = 230 days [114.5–361.5]) compared to the CD group (median = 355 days [212.5–562.5]; *P *< 0.0001).

**Figure 2:**
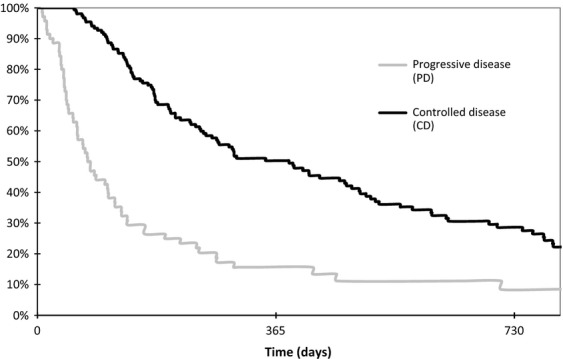
Overall survival according to status of disease after the 45th day of epidermal growth factor receptor tyrosine-kinase inhibitors treatment (*n *=* *227 patients). PD, progressive disease; CD, controlled disease. Kaplan–Meier method (*P *<* *0.0001) and the log-rank test. Controlled disease group: median of 385 days, 95% CI [267–481], 1st–3rd quartiles [169–776]; PD group: median of 77 days, 95% CI [61–109], 1st–3rd quartiles [41–195].

A total of 137 patients had cutaneous toxicity (57.3%). This was less frequent in the PD group (*n *= 29, 38.7%) compared to the CD group (*n *= 108, 65.9%, *P *< 0.0001). Progression-free survival was 29 days in the PD group (95% CI 27–32). It was 115 days in the CD group (95% CI 97–154). Median OS was shorter in the PD group (77 days, 95% CI 61–109; *P *< 0.0001) than in the CD group (385 days, 95% CI 267–481; Fig. 2).

### Multivariate analyses

Seven factors were associated with early progression in the univariate analyses: age (odds ratio [OR] 0.96, 95% CI [0.93–0.98], *P *= 0.005), current smoking (OR 3.99, 95% CI [1.69–9.40], *P *= 0.002), PS ≥ 2 (OR 2.11, 95% CI [1.76–3.80], *P *= 0.012), weight loss ≥10% (OR 2.42, 95% CI [1.10–5.31], *P *= 0.028), abdominal metastasis (OR 2.22, 95% CI [1.22–4.03]; *P *= 0.009), nos-NSCLC (OR 2.91, 95% CI [1.52–5.56]; *P *= 0.001), and time since diagnosis (OR 0.99, 95% CI [0.99–0.99]; *P *= 0.001) (Table 3). Biomarkers were not included into the logistic regression model because of the amount of missing data. Toxicity was not included in the logistic regression model because we were looking for factors that could be evaluated before the beginning of treatment.

**Table 3:** Factors predictive for early progression in multivariate analyses (*n *=* *194 patients)

	Odds ratio	95% CI	*P*
Current smoking	2.276	0.860–6.025	0.098
Performance status ≥ 2	1.843	0.947–3.584	0.072
Abdominal metastatic site	2.174	1.125–4.199	0.021
Nos-NSCLC	1.978	0.921–4.251	0.080

Logistic regression. Biomarkers and weight loss were not included in the model because of the amount of missing data. Age and time since diagnosis were not included because of their lack of clinical implication.

Because of the limited number of events, factors with an OR near 1 (age and time since diagnosis) were excluded from the multivariate analysis. Weight loss was judged redundant with PS, and was excluded as well. In multivariate analysis, abdominal metastases were the only factor associated with early progression with an OR of 2.17 (95% CI [1.12–4.19]; *P *= 0.021).

## Discussion

The choice of the best second or third-line treatment in NSCLC, that is, chemotherapy or EGFR-TKIs, is a frequent issue. This study focuses on potential factors associated with early progression in a Caucasian cohort of patients treated with EGFR-TKI.

We defined early progression as progression before the 45th day of treatment. This threshold was based on the PFS of patients with wild-type *EGFR* NSCLC receiving EGFR-TKI. In previous studies, median PFS has been about 2 months (2.4 months in the recent TAILOR study 6). Thus, to enable assessment of early progression, our threshold needed to be lower. The time of the first carcinological assessment varied in our cohort, but took place before the 45th day.

Median OS was 8.0 months (242 days) while only 6.7 months (203 days) in the BR 21 study 12. This difference can be explained by the fact that our patients belonged to a “real life” cohort, which means that they had been selected by physicians. In the Tarceva Lung Cancer Survival Treatment, a large phase IV open-label study 24, OS was 7.9 months (240 days), close to the value observed in this study. In the CD group of this study, median OS was even longer (385 days). Thus, identifying predictive factors of early progression would be a strategy to improve management of EGFR-TKIs after failure of platin-based chemotherapy in *EGFR* wild-type patients.

In this cohort, younger age, current smoking, PS ≥ 2, weight loss ≥10%, shorter time since diagnosis, pathological classification as non-otherwise-specified NSCLC, and the presence of abdominal metastasis were associated with early progression of NSCLC with EGFR-TKI therapy. In multivariate analysis, abdominal metastases were the only factor associated with early progression.

In this study, *KRAS* analyses were not conclusive as only 3.8% of the tumors were *KRAS-*mutated. This proportion of *KRAS*-mutated tumors is lower than in previous studies, due to the facts that the cohort included all pathological types and that the paraffin-embedded samples were aged of more than 3 years 25. Whether *KRAS* mutations are associated with early progression under EGFR-TKIs is still debated 26–30. In the TAILOR study comparing docetaxel versus erlotinib 6, *KRAS-*mutation status was not associated with a reduced time to progression during EGFR-TKI therapy. *EGFR* mutations were detected in 7.9% of Caucasian patients and no EGFR mutation was detected in patients with early progression.

Factors associated with early progression under EGFR-TKI might be predictive of EGFR-TKI resistance, or prognostic, related to tumor aggressiveness. Weight loss and poor PS seem to be prognostic rather than predictive factors 31. Current smoking might be a predictive factor. It was associated with early progression, with no difference between nonsmokers and ex-smokers. Tobacco is an inducer of the p450 cytochrome and can reduce erlotinib plasma concentrations in current smokers 32. Whether increasing the dose of erlotinib may be an option, is still debated 33.

In this study, classification as nos-NSCLC was associated with early progression under EGFR-TKI. Reduced response rate and OS under EGFR-TKI have already been shown in large-cell and nos-NSCLC 34,35. It is admitted that EGFR expression is lower in nos-NSCLC. This might explain a resistance to EGFR-TKI in this subtype 36. However, nos-NSCLC histology might also be a prognostic factor. Indeed, a study based on adenocarcinomas 37showed that poorly differentiated adenocarcinomas were associated with shorter OS. It should be noted that the number of nos-NSCLC in our cohort was high. This could be accounted for by the fact that about 85% of diagnosis were made on small-sized histological samples. The number of squamous-cell lung cancer was comparatively low (16.3% in this study vs. 24% in the large phase IV study TRUST 24). This reflects the fact that this is a retrospective study, thus patients were selected by clinicians who were probably more reluctant to prescribe EGFR-TKI in squamous cell lung cancer than in adenocarcinoma.

In multivariate analysis, abdominal metastases were the only factor associated with early progression. Abdominal metastasis remain associated with early progression even after removing Asian patients (*P* = 0.008, data not shown) or patients with known *EGFR* mutation (*P* = 0.012, data not shown). Several cohort-based studies have shown a correlation between abdominal metastases and shorter OS or PFS under EGFR-TKIs 38,39. No physiopathological hypothesis was drawn up in these studies. Does the presence of abdominal metastasis correspond to an aggressive pattern of cancer, as presence of liver abdominal is usually associated with poor prognosis 31? Or does the presence of abdominal metastasis correspond to a specific phenotype with a primary resistance to EGFR-TKI? In our study, none of the 19 patients with a known *EGFR* mutation had any abdominal metastases, whereas there were 21 patients with abdominal metastasis in the *EGFR* wild-type subgroup (*P* = 0.016), but no such association has been reported before in the literature. On the contrary, in a publication studying association between oncogenes and patterns of metastatic spread 40, 10 among 39 patients with *EGFR*-mutated tumors had liver metastasis. Thus, the significance of the association between abdominal metastasis and early progression during EGFR-TKI treatment remains to be determined. It would also be interesting to know if this result is “driven” by a particular site of abdominal metastasis such as liver for example.

To conclude, the presence of abdominal metastasis was the only independent factor associated with early progression during EGFR-TKI therapy in a real-life cohort of NSCLC patients. Whether abdominal metastases are predictive of EGFR-TKI resistance remains uncertain.

## Supporting Information

**Table S1.** Pathological subtypes and mucosecretion in patients with adenocarcinoma (*n* = 134). **Table S2.** Metastatic abdominal sites in patients with abdominal metastasis (*n* = 71).
