# Supplementary material for: Factors associated with early progression of non-small-cell lung cancer treated by epidermal growth factor receptor tyrosine-kinase inhibitors
Source: Cancer Med. 2014 Jan 10;3(1):61–9. doi: 10.1002/cam4.180 (PMC3930390; doi:10.1002/cam4.180)
Supplement: Supplementary file 1 — Table S1. Pathological subtypes and mucosecretion in patients with adenocarcinoma (n = 134). Table S2. Metastatic abdominal sites in patients with abdominal metastasis (n = 71). [file cam40003-0061-sd1.docx]

**Table S1: Pathological subtypes and mucosecretion in patients with adenocarcinoma (*n*=134).**

|  | **Overall population (*n*=134) (%)** | **PD group (*n*=36) (%)** | **CD group (*n*=98) (%)** | ***p*** |
| --- | --- | --- | --- | --- |
|  |  |  |  |  |
| **Predominant pattern^1^** |  |  |  |  |
| - ***Acinous*** | 28 (20.9) | 6 (16.7) | 22 (22.4) | **0.925** |
| - ***Lepidic*** | 21 (15.7) | 5 (13.9) | 16 (16.3) |  |
| - ***Papillary*** | 11 (8.2) | 3 (8.3) | 8 (8.2) |  |
| - ***Solid*** | 9 (6.7) | 3 (8.3) | 6 (6.1) |  |
| - ***Unknown*^2^** | 65 (48.5) | 19 (52.8) | 46 (46.9) |  |
| **Muco-secretion^1^** |  |  |  | **0.743** |
| - ***yes*** | 17 (12.7) | 5 (13.9) | 12 (12.2) |  |
| - ***no*** | 43 (32.1) | 10 (27.8) | 33 (33.7) |  |
| - ***unknown^2^*** | 74 (55.2) | 21 (58.3) | 53 (54.1) |  |

1: Chi-squared test; 2: missing data have been suppressed for the statistical analyses.

**Table S2: Metastatic abdominal sites in patients with abdominal metastasis (n=71)**

|  | **Overall population (*n*=71)** | **PD group (*n*=31)** | **CD group (*n*=40)** | ***p*** |  |
| --- | --- | --- | --- | --- | --- |
| **Liver^1^** | 31 (14.2%) | 14 (20.3%) | 17 (11.4%) | **0.081** |  |
| **Adrenal^1^** | 33 (15.1%) | 13 (18.8%) | 20 (13.4%) | **0.299** |  |
| **Adenopathy^1^** | 11 (5%) | 6 (8.7%) | 8 (3.3%) | **0.091** |  |
| **Peritoneum^2^** | 2 (0.9%) | 2 (2.9%) | 0 (0%) | **0.098** |  |
| **Pancreas^2^** | 1 (0.5%) | 0 (0%) | 1 (0.7%) | **1.0** |  |
| **Kidney^2^** | 3 (1.4%) | 0 (0%) | 3 (2.0%) | **0.553** |  |
| **Spleen^2^** | 1 (0.5%) | 0 (0%) | 1 (0.7%) | **1.0** |  |

1: Chi-squared test; 2: Fisher’s exact test
